# Pupil dilation tracks the dynamics of mnemonic interference resolution

**DOI:** 10.1038/s41598-018-23297-3

**Published:** 2018-03-19

**Authors:** Roger Johansson, Philip Pärnamets, Amanda Bjernestedt, Mikael Johansson

**Affiliations:** 1 Department of Psychology, Lund University, Lund, Sweden; 2 Department of Philosophy and Cognitive Science, Lund University, Lund, Sweden; 3 Department of Clinical Neuroscience, Karolinska Institutet, Karolinska, Sweden

**Keywords:** Neurophysiology, Human behaviour

## Abstract

Mnemonic interference refers to the inability to retrieve a goal-relevant memory due to interference from goal-irrelevant memories. Understanding the causes of such interference and how it is overcome has been a central goal in the science of memory for more than a century. Here, we shed new light on this fundamental issue by tracking participants’ pupil response when they encode and retrieve memories in the face of competing goal-irrelevant memories. We show that pupil dilation systematically increased in accordance with interference from competing memory traces when participants retrieved previously learned information. Moreover, our results dissociate two main components in the pupillary response signal: an early component, which peaked in a time window where the pupillary waveform on average had its maximum peak, and a late component, which peaked towards the end of the retrieval task. We provide evidence that the early component is specifically modulated by the cognitive effort needed to handle interference from competing memory traces whereas the late component reflects general task engagement. This is the first demonstration that mnemonic interference resolution can be tracked online in the pupil signal and offers novel insight into the underlying dynamics.

## Introduction

The ability to encode new information and to be able to retrieve it at a later stage is fundamental for human adaptive behavior, yet we frequently fail to recall sought-after information. A major cause of such retrieval failure is interference from other information also active in memory^1,2^. Typically, proactive interference (PI) from previously learned information impairs memory for more recently acquired one^3^. While it is well established that the probability of retrieving an item declines with the number of previously studied items^4,5^ and that response latencies increase in accordance with the set-size of those items^6^, it has proven challenging to directly capture the presence of PI, as well as how and when it is overcome. A prominent explanation is that, when sharing the same retrieval cue, interference from prior memories reduces the discriminability of more recent ones at the retrieval stage^2,6,7^. This account is consistent with the cue-overload principle^5^ and recent studies further suggest that cognitive control processes of selection and inhibition, as mediated by frontal lobe mechanisms, are crucial for resolving competition under such circumstances^8–16^. However, PI can also be attributed to the encoding stage and it has been suggested that competition from previously studied items may increase memory load and inattention to more recent items, leading to less complete encoding and thus a reduction in later retrieval^4,17,18^.

To shed further light on these issues, the present study employed pupillometry in an experiment where PI successively builds up and then gets released. Previous research has established that variations of pupil size can be used as a reliable online measure of “cognitive effort”^19,20^ or usage of attentional resources^21^. For instance, the pupil has been shown to dilate in accordance with attentional control demands^22^, increased response conflict^23,24^ and retrieval effort^25^. Based on such findings, we expect the pupil signal to be a particularly useful biomarker when studying mnemonic interference and we predict that it should reflect the dynamics of how attentional resources are recruited and released for participants who encode and retrieve items in the face of PI. While overall pupil size during encoding has been inconclusively explored in an early study of PI^26^, this is the first study to systematically investigate phasic pupil size changes in all stages of competitive memory retrieval.

## Results

**Figure 1:**
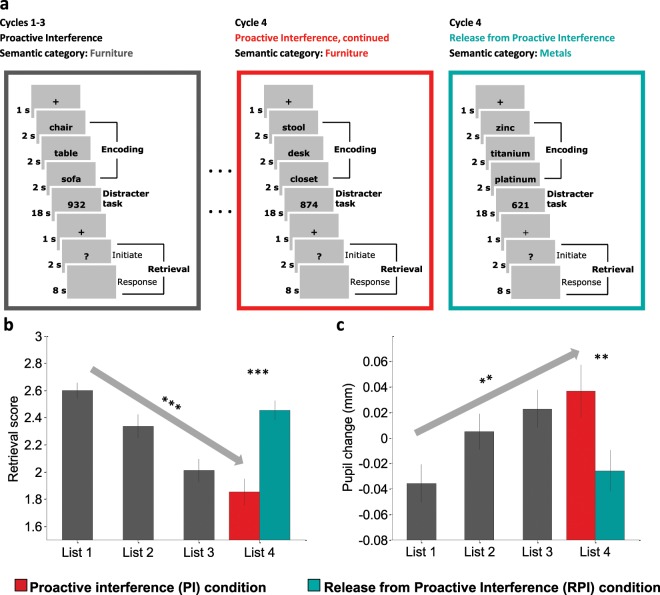
Experimental Design, Memory Performance and Pupillary Changes. (**a**) Participants encoded 3 words from the same semantic category, engaged in a distracter task and were in a retrieval phase to orally recall the 3 words. This procedure was repeated over 4 cycles. Retrieval commenced with an initiation phase and a response phase. The initiation phase was indicated by a “?” in the center of the screen and the response phase was indicated with a speaker symbol in the center of the screen. Participants were not allowed to respond until the response phase was initiated. The distracter task was to orally count backwards in steps of 7 from a randomly generated three-digit number. In the fourth and final word list, proactive interference from previous word lists continued to build up in the PI condition (middle panel), whereas a release from proactive interference was created by changing the semantic category in the RPI condition (right panel). The semantic categories “furniture” and “metals” are here used to illustrate a possible cycle for both PI and RPI conditions, whereas in the actual experiment all categories were used equally often in all conditions across participants. (**b**) Average retrieval score for word lists 1–3 and for the fourth and final word list when separated into PI and RPI conditions. (**c**) Average pupil change in mm (compared to baseline) for word lists 1–3 and for the fourth and final word list when separated into PI and RPI conditions. Error bars denote SEM, **p < 0.01, ***p < 0.001.

Our experiment was an adapted version of the release from proactive interference paradigm^4^, in which participants encode and retrieve three words from the same semantic category in three consecutive cycles, leading to a progressive build-up of proactive interference from previous word lists. In a fourth and final cycle, two experimental conditions are created: proactive interference (PI) and release from proactive interference (RPI). In the PI condition, proactive interference continues to build up when participants encode and retrieve another word list from the same semantic category, whereas in the RPI condition a release from proactive interference is created by changing the semantic category of the final word list (see Fig. 1a). As expected, a repeated measures ANOVA revealed that retrieval performance systematically declined across the first three word lists and continued to decline in the fourth and final word list for the PI condition (linear contrast, *F*(1, 29) = 143.757, *P* < 0.001, *η*^2^ = 0.83, see Fig. 1b and Supplementary results S1 for post-hoc comparisons between all the word lists). Consistent with expectations, a release from proactive interference was revealed in the final word list for the RPI condition (Fig. 1b). On average, participants retrieved 2.45 words (SEM = 0.07) in the RPI condition and 1.85 words (SEM = 0.10) in the PI condition (*t*(29) = 9.10, *P* < 0.001, *d* = 1.66). These results confirm that interference systematically built up in accordance with the number of competitors from previous word lists and that our manipulation to eliminate such interference during the last word list in the RPI condition was successful.

### Pupil dilation and mnemonic interference

To test the hypothesis that changes in the pupil signal capture mnemonic interference, we analyzed the average change in pupil diameter during word list retrieval in the four cycles. A repeated measures ANOVA revealed a pattern that mirrored the retrieval performance, where the pupil diameter systematically increased across the first three word lists and continued to increase in the fourth and final word list for the PI condition (linear contrast, *F*(1, 29) = 11.874, *P* = 0.002, *η*^2^ = 0.29, see Fig. 1C and Supplementary Results S2 for post-hoc comparisons between all the word lists). Critically, the pupil diameter decreased when proactive interference was released in the final word list for the RPI condition (Fig. 1c). On average, pupil diameter differed by 0.062 mm (SEM = 0.004) between PI and RPI conditions (*t*(29) = 3.28, *P* = 0.003, *d* = 0.60). To test if interference was reflected in the pupil signal already during learning, we conducted corresponding analyses also when the word lists were encoded. While pupil diameter overall decreased across word lists (linear contrast, *F*(1, 29) = 9.736, *P* = 0.004, *η*^2^ = 0.25, see Supplementary Fig. S1 and Supplementary result S3 for post-hoc comparisons between all the word lists), no significant difference in pupil diameter emerged between PI and RPI conditions (*P* = 0.35, see Fig. S1). The finding that the pupil size decreased across word list encoding, without a difference between interference conditions in the fourth and final word list, parallels an early exploration by Engle^26^ and suggests that this effect is sensitive to task familiarity rather than interference from previous word lists.

In sum, these results demonstrate that pupillometry reliably tracks interference from competing memory traces and that accompanying retrieval failure results from processes operating at retrieval rather than encoding. However, the reported pupillary effects can be attributed either to the response conflict that arises when a sought-after memory trace is to be selected against its competitors or to the cognitive control mechanisms recruited to handle such interference. To disambiguate between those two alternatives, we conducted additional analyses, where we first targeted the temporal dynamics in the pupillary response signal and then addressed how it relates to retrieval success and mnemonic mechanisms.

### Temporal dynamics in the pupillary response signal

**Figure 2:**
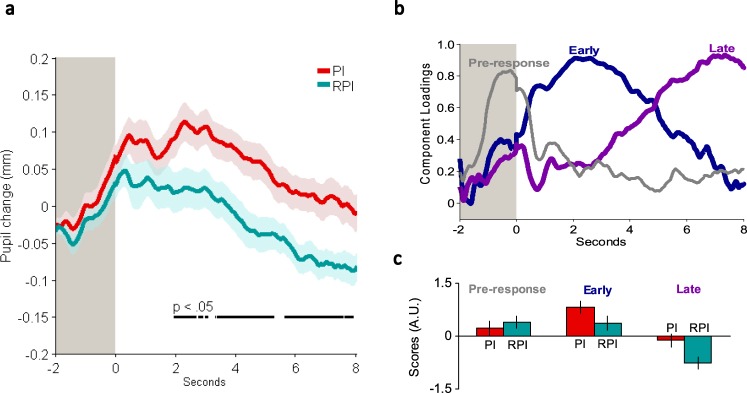
Temporal dynamics of pupillary changes. (**a**) Aggregated trial-locked pupil change in mm during retrieval of the fourth and final word list for PI and RPI conditions. The shaded grey area to the left in the figure represents the initiation phase (2 seconds), i.e. before participants were allowed to respond. Shaded areas for both PI and RPI conditions denote SEM. Black lines in the lower half of the figure denote significant time points (*P* < 0.05) by permutation tests, corrected for multiple comparisons. (**b**) Principal component analysis (PCA) on the pupil data. Displayed are standardized component loadings for 3 components accounting for 81.3% of the variance: Pre-response Component (11.0%), Early Component (41.2%) and Late Component (29.1%). (**c**) Component scores (arbitrary units) for the three components. Error bars denote SEM.

To further specify the pupillary dynamics during retrieval of the final word list over PI and RPI conditions, we conducted a time-series analysis using permutation tests on the per participant and condition average pupil signal. These analyses revealed that the PI and RPI conditions started to differ significantly 1.9 seconds into the response phase (*P* < 0.05), and in principle continued throughout the entire response phase (Fig. 2a, See Supplementary analysis S3 and Supplementary Fig. S1 for a corresponding time-series analysis during encoding). However, aggregated pupil signals in time-series analyses are likely to average across asynchronous peaks of cognitive processing with the consequence of purging less sustained, more phasic responses^23,27–29^.

To identify onset, offset, and duration of different processes contributing to the morphology of the aggregated waveform, a temporal principal component analysis (PCA) was computed on the averaged trial-locked pupil signal per participant and condition averages, thus treating every data point (each sample) in time as a dependent variable. A scree plot indicated that three components, accounting for most of the variance, were to be retained: (Fig. 2b): a Pre-response Component (peaking during the initiation phase), an Early Component (peaking around 2–3 seconds into the response phase, i.e. in the time window where the pupillary waveform on average had its maximum peak) and a Late Component (peaking towards the end of the response phase). Their component scores were then compared over conditions. Both the Early and the Late Components differed significantly between PI and RPI conditions (Early Component, *t*(29) = 2.65, *P* = 0.013, *d* = 0.49; Late Component 2, *t*(29) = 3.99, *P* < 0.001, *d* = 0.73; see Fig. 2c). The Pre-response Component did not differ significantly between conditions (*P* = 0.30). Given that the pupil reacts rather slowly, this peak is likely to reflect perceptual and attentional processes in response to the initiation phase and anticipation of engaging in the retrieval task^27,29^, which is not expected to be dependent upon differences between the two interference conditions.

### Pupillary components and mnemonic mechanisms

To relate the Early and the Late pupillary components to mnemonic mechanisms, we first calculated a mnemonic interference index based on the average retrieval performance of the fourth and final word list for each participant [(RPI−PI)/RPI)]^8^. For both the Early and the Late Component, we then correlated this index with a pupillary difference score between RPI and PI conditions for their respective component scores (RPI−PI). Those correlations thus represent the relationship between the degree of mnemonic interference and the magnitude of the pupillary difference between RPI and PI conditions.

**Figure 3:**
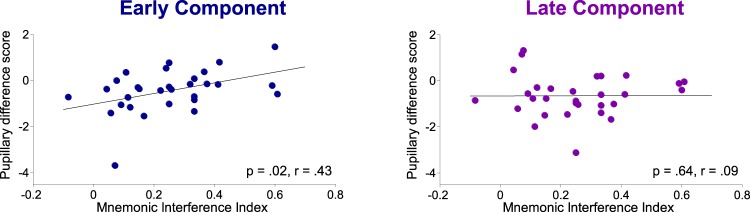
Correlations between mnemonic interference indexes and pupillary difference scores. Correlations between participants’ mnemonic interference index (RPI−PI)/RPI and their respective pupillary difference scores (component scores) between RPI and PI conditions (RPI−PI) when retrieving the final (fourth) word lists for both the Early and the Late Component.

A positive correlation emerged for the Early Component, *p* = 0.02, *r* = 0.43, but not for the Late Component, *p* = 0.64, *r* = 0.09, indicating that participants with a lower interference index, i.e. those who were better at handling the interference, were those who showed larger pupil dilation in the Early Component during PI conditions relative to RPI conditions, and vice versa (Fig. 3). With the caveat that you need to be very cautious when interpreting correlations with rather few data points, the results suggest that the Early Component is modulated by cognitive control mechanisms recruited to handle mnemonic interference whereas the Late Component is not directly modulated by interference.

To further scrutinize this interpretation, we categorized all trials according to retrieval success: Successful (all three words retrieved) or Unsuccessful (only one or no words retrieved). Using those categories, we then reran the PCA over PI and RPI conditions. Comparable components as in the previous PCA emerged (Pre-response, Early and Late Components), peaking in similar time windows (see Supplementary Fig. S2).

**Figure 4:**
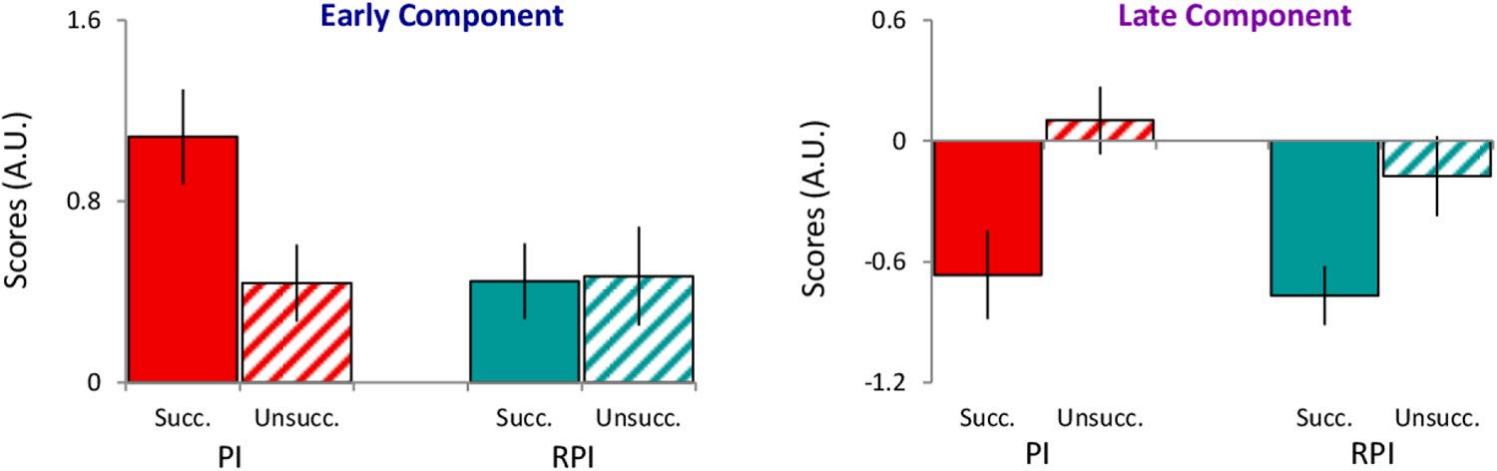
Pupillary component scores when splitting trials into Successful and Unsuccessful retrieval. Pupillary component scores for PI and RPI conditions when splitting trials into Successful (all 3 words retrieved) and Unsuccessful retrieval (1 or 0 words retrieved). Error bars denote SEM. In cases where participants did not have any Successful or Unsuccessful performance trials, those participants were excluded, which resulted in the following number of participants: PI-High (n = 27), RPI-High (n = 30), PI-Low (n = 29), RPI-Low (n = 20).

Results revealed that scores for the Early Component were significantly larger during successful PI retrieval compared to both unsuccessful PI retrieval, *t*(25) = 2.70, *p* = 0.012, *d* = 0.53, and successful RPI retrieval, *t*(26) = 2.77, *p* = 0.010, *d* = 0.53. Due to unsuccessful RPI retrieval being rare, only few participants could be included when comparing that condition with successful PI retrieval (n = 16), and while not significant, *t*(16) = 1.29, *p* = 0.21, *d* = 0.31, the overall pattern was consistent with the previous comparisons (see Fig. 4). No significant differences were found between any other combinations (all *p*’s > 0.59). Taken together, these results corroborate the interpretation that the Early Component represents a pupillary signature of the cognitive control needed to resolve interference from competing memories.

Results for the Late Component scores were significantly larger for unsuccessful PI retrieval than for successful PI and RPI retrieval, *t*(25) = 2.81, *p* = 0.010, *d* = 0.55; *t*(28) = 3.98, *p* < 0.001, *d* = 0.74. Again, due to unsuccessful RPI retrieval being rare, only few participants could be included when comparing that condition with successful PI and RPI retrieval (n = 16; n = 19) and while not significant, *t*(16) = 1.23, *p = *0.24, *d* = 0.30; *t*(19) = 1.41, *p* = 0.18, *d* = 0.32, the overall patterns were consistent with the previous comparisons (see Fig. 4). No significant differences was found between successful PI and RPI retrieval, *t*(26) = 1.49, *p = *0.15, *d* = 0.33, nor between unsuccessful PI and RPI retrieval, *t*(18) = 1.42, *p* = 0.17, *d* = 0.29. Taken together, these results show that the Late Component is associated with retrieval success, independent of mnemonic interference. Given the negative component scores during retrieval success, it appears that associated attentional resources get released once the task objective is completed, whereas such resources remain sustained during unsuccessful retrieval, likely due to continued retrieval attempts^25^ (see Fig. S2a in the online Supplemental Materials). It would thus be the maintenance and termination of such task engagement that gets reflected in the Late Component.

## Discussion

In the present study, we investigated the relationship between pupil dilation and mnemonic interference during competitive memory retrieval. Our main finding was that pupil size systematically increased as a function of demands to resolve proactive interference (PI) during retrieval of previously learned information. While previous research has attributed PI, at least partly, to how readily something was encoded^4,17^, we found no support for such claims in the present study. Instead our results add weight to accounts where PI is considered to disrupt the retrieval process, i.e. when a memory needs to be selected against a set of competitors sharing the same retrieval cue^5,6,9^. Moreover, by applying a temporal PCA we identified two main components in the pupillary response signal: an early component, which peaked in a time window where the pupillary waveform on average had its maximum peak, and a late component, which peaked towards the end of the retrieval task. By associating those two components with retrieval performance, we were able to further specify our pupillary main result in relation to mnemonic interference resolution and to dissociate its influence from mere task engagement. Critically, the early component was found to be specifically modulated by the cognitive control needed to successfully handle PI resolution whereas the late component was associated with retrieval success, independent of mnemonic interference, suggesting that this component reflects maintenance and termination of general task engagement.

While previous work has captured the buildup and release of PI with a measure of recall latency^6^, the pupillary response signal provides some major advantages: it is a continuous online measure, which over time is capable of tracking the buildup of interference, as well as if and when such interference gets resolved. Moreover, the pupil signal can be registered independent of overt responses. Since the present study did not measure response times, we do not know exactly how pupillary changes relate to recall latencies. While this is an important aspect to consider, we want to underscore that response time differences between PI and RPI conditions cannot explain our main finding involving the early PCA component and interference control. Since previous research has established that response latencies increase in accordance with the buildup of PI^6^, successful retrieval during PI trials will have larger response latencies than successful retrieval during RPI trials. Thus, if such response time differences would modulate the reported PCA-result during successful retrieval, we would expect pupillary differences between PI and RPI trials to occur in the later time window, which is contrary to our results (See Figs 2, 4 and S2).

Exactly how the pupil signal is related to the underlying cognitive processes is a complex issue. On a neurobiological level, pupillary changes associated with cognitive processing are tightly linked with the release of norepinephrine (NE) from the locus coeruleus (LC)^30–32^. The LC-NE system is known to affect cognitive control systems in prefrontal cortex (PFC)^33,34^ and there is evidence that projections from the anterior cingulate cortex (ACC), a structure highly sensitive to the occurrence of conflict^35,36^, simultaneously drive LC activity and increased responsivity in PFC structures^30,37,38^. Considering that interference from competing memories has been demonstrated to activate the ACC^10,11^, and its resolution has been shown to be modulated by cortical structures in PFC^8,9^, such results provide a plausible neurobiological link between pupillary changes and mnemonic interference resolution. Moreover, there is much evidence that mechanisms recruited to overcome mnemonic interference entail PFC-mediated retrieval inhibition^8,14–16^ and it is thus conceivable that the reported pupillary effects reflect the cognitive effort involved in recruiting such inhibitory control mechanisms.

To conclude, the present study shows that pupillometry can track mnemonic interference resolution, which is fundamental for successful memory retrieval in the face of competition, and demonstrates the possibility to observe and study the underlying dynamics using a non-invasive online measure. It further identifies a pupillary signature for the cognitive control needed to overcome mnemonic interference in the early PCA component, suggesting a critical time window for further investigation.

## Methods

### Participants

Thirty-three healthy adults participated in the experiment and were compensated with a cinema voucher. Three were removed owing to extensive data loss and technical problems, leaving thirty participants (15 females; mean age 27, SD 8.5). The participant sample size follows the number of participants used by similar studies in the field. All were fluent in Swedish and had normal or corrected-to-normal vision. All methods were conducted in accordance with the Swedish Act concerning the Ethical Review of Research involving Humans (2003:460). The Ethics Committee at the Department of Psychology, Lund University, has corroborated that the present research protocol follows the research ethics guidelines established by Swedish authorities. Participants gave written informed consents.

### Design and stimuli

We applied pupillometry in an adapted version of the release from proactive interference paradigm^4^. Participants were to encode and retrieve three words from the same semantic category in three consecutive cycles, leading to a progressive build-up of proactive interference from previous word lists. In a fourth and final cycle, two experimental conditions were then created: proactive interference (PI) and release from proactive interference (RPI). In the PI condition, proactive interference continues to build up when participants encode and retrieve another word list from the same semantic category, whereas a release from proactive interference is created by changing the semantic category of the final word list in the RPI condition. In total, participants encoded and recalled 96 different word lists: 24 blocks of four consecutive word lists, whereof the fourth and final word list was either a PI or a RPI condition (12 blocks of PI; 12 blocks of RPI).

For the ordering of conditions, semantic categories and word stimuli, a unique version of the experiment was created for each participant in order to avoid order and learning effects. The order of PI- and RPI-conditions was pseudorandomized within each version, meaning that the order was random with the restriction that each condition never occurred more than three times in a row. The word stimuli were selected from the Swedish category norms^39^ and consisted of 36 semantic categories (See Supplementary materials S4) with 12 words in each category (a total of 432 words). The categories were selected to have as few semantic attributes as possible in common with each other. To increase mnemonic interference from previous words lists they were presented with decreasing semantic typicality, where the first word list always consisted of the three most typical words from their semantic category and the final (fourth) word list always consisted of the three least typical words from their semantic category.

To control for order and semantic category effects several precautions were taken. In a first step, the categories were organized into three sets, A, B and C, with 12 categories in each. These sets were then organized into a Latin square. For each experiment version, one row of the Latin square was used; going back to the first after every combination had been used. The first letter of each combination denoted the set of categories that would be used for the three preceding word lists (word lists 1–3) and the fourth and final word list in a PI condition. The second letter denoted the set of categories that would be used for the three word lists preceding a RPI condition, and the third letter denoted the set of categories that would be used for the fourth and final word list in a RPI condition. For example, experiment version 1 used the combination ABC. In that version the categories from set A were used for the three preceding word lists (word lists 1–3) and the fourth and final word list in the PI conditions. Set B were used for the three word lists (word lists 1–3) preceding the RPI conditions, and set C for the fourth and final word list in the RPI conditions. The internal order in which the categories appeared within each set was shuffled after every third experiment version. Importantly, all lists were thus used equally often in the PI and RPI conditions, eliminating any category specific effects on the results.

Free recall was used during the retrieval phase, where participants responded orally. This procedure enabled participants to keep their gaze at a fixed point throughout the entire retrieval phase, while still being able to respond, which is critical when pupil data is gathered.

### Data Acquisition

Pupil data was recorded using a SensoMotoric Instruments (SMI RED) eye tracker, running iView X 2.7 software and sampling at 250 Hz. In this setup the eye tracking camera is located centrally under the presentation monitor at a distance of 70 cm from the participant. The eye tracker is developed for a contact-free pupil measurement with automatic head-movement compensation, in a range of 40 × 20 cm at a distance of 70 ± 10 cm. In effect, movements toward and away from the camera do not confound measured pupil size. The system measures pupil diameter in mm for both pupils and can according to the manufacturer (SMI) detect changes as small as 0.004 mm. This contact-free setup thus offers high quality data while allowing verbal responses to be collected. A Dell Optiplex 755 PC presented stimuli using PsychoPhysics Toolbox^40,41^ in MATLAB 2012b on a 22′′ monitor with a resolution of 1024 × 768 pixels. Task stimuli were presented in a black Arial 14 point font on a grey background (RGB: 125,125,125). Calibration and validation of gaze data was conducted prior to each participant’s experimental session and was repeated until the deviation scores were below an error of 0.5° both horizontally and vertically.

### Data reduction and pre-processing of pupil data

Collected data was pre-processed using in-house software written in Matlab 7.4. First, to correct for blinks and missing data all samples of zero data was discarded, including the 10 preceding samples and the 10 following samples. Second, to correct for physiologically implausible values, samples with data below 1 mm or above 9 mm were also discarded. Third, linear interpolation was performed to correct the data for those exclusions, applying an algorithm based on the last good data point and the first good data point. Trials where more than 50% of the samples had to be excluded were discarded from analyses (4.5% of the trials were discarded). Fourth, to reduce high frequency noise and smooth the data it was filtered using a 10 Hz low-pass filter. Finally, to correct for physiologically implausible dilations or constrictions, samples which compared to the previous sample increased with a velocity above 1 mm/sec or decreased with a velocity below −4.0 mm/sec were discarded. Linear interpolation was then performed to correct for those exclusions.

For each retrieval trial, the average pupil diameter of the last 200 ms from the baseline screen was subtracted from each sample (see Fig. 1a). Averaged stimulus-locked pupillary responses were then computed for each participant and each condition (PI and RPI) over the entire retrieval phase (10 sec) for word lists 1–4. For the encoding phase the same logic was used, where the last 200 ms from the screen preceding the onset of the first word was subtracted from each sample (following onset of the first word). Averaged stimulus-locked pupillary responses were then computed for each participant and each condition (PI and RPI) over the entire encoding phase (6 sec) for word lists 1–4.

### Data analyses

Data were analyzed using paired *t*-test (two-tailed) and analyses of variance with repeated measures. Post-hoc comparisons used paired *t*-tests and Bonferroni correction for multiple comparisons. The Mauchly’s test was applied to verify the assumption of sphericity.

For the time-course analyses we conducted permutation analyses (20 000 permutations) per participant and condition (PI and RPI) averages during retrieval of the (fourth) last word list. To control for multiple comparisons in time series data, significant differences between conditions were determined using the *t*_max_ method introduced by Blair and Karniski^42^.

### Principal Component Analyses (PCA)

A temporal principal component analysis (PCA) was computed on the averaged stimulus-locked pupil signal per participant and condition averages during retrieval of the fourth and final word list, treating every sample (2500 data points) in time as a dependent variable. A Varimax rotation was then performed on components characterized by an eigenvalue equal to or greater than the average variance of the original variables. Each extracted component represents a group of data points that correlate highly among themselves, but not with another group of data points. Following Kayser and Tenke^43^ an unrestricted PCA using the unstandardized covariance matrix with Kaiser normalization and Varimax rotation was used on all 2500 unrestricted components to produce maximal component loadings on one component and minimal on others. Component loadings are correlations that define the relationship between a variable and a component, thus showing the rise, peak and fall of loadings on each component (see Fig. 2b and Supplementary Fig. S2). Importantly, component loadings do not discriminate between conditions and can thus be used as weights to compute component scores for both PI and RPI conditions. Component scores thus represent the relative contribution (weight) of each loading pattern for each condition (see Figs 2c and 4).

### Data availability

The datasets generated during and/or analysed during the current study are available from the corresponding author on request.

## Electronic supplementary material

Supplementary information
