# Publisher Correction: Pupil dilation tracks the dynamics of mnemonic interference resolution

**DOI:** 10.1038/s41598-019-55183-x

**Published:** 2020-01-10

**Authors:** Roger Johansson, Philip Pärnamets, Amanda Bjernestedt, Mikael Johansson

**Affiliations:** 1 Department of Psychology, Lund University, Lund, Sweden; 2 Department of Philosophy and Cognitive Science, Lund University, Lund, Sweden; 3 Department of Clinical Neuroscience, Karolinska Institutet, Karolinska, Sweden

Correction to: *Scientific Reports* 10.1038/s41598-018-23297-3, published online 19 March 2018

In the original version of this Article, the error bars in graphs b and c of Figure 1 were omitted.

In addition, the ORCID ID for Mikael Johansson was omitted.

These errors have now been corrected in the HTML and PDF versions of the Article.

