# Supplementary material for: Pupil dilation tracks the dynamics of mnemonic interference resolution
Source: Sci Rep. 2018 Mar 19;8:4826. doi: 10.1038/s41598-018-23297-3 (PMC5859100; doi:10.1038/s41598-018-23297-3)
Supplement: Supplementary file 1 — Supplementary information [file 41598_2018_23297_MOESM1_ESM.pdf]

Supplementary information

Pupil dilation tracks the dynamics of mnemonic interference resolution

Roger Johansson[1*], Philip Pärnamets[2,3], Amanda Bjernestedt[1] and Mikael Johansson[1]

[1]Department of Psychology, Lund University, Sweden

[2]Department of Philosophy and Cognitive Science, Lund University, Sweden

[3]Department of Clinical Neuroscience, Karolinska Institutet, Sweden

* Correspondence concerning this article should be addressed to Roger Johansson, Department of Psychology, Lund University, Box 213, SE-221 00 Lund, Sweden, email: roger.johansson@psy.lu.se

Supplementary results

S1. *Retrieval scores*

A one-way repeated-measures ANOVA was conducted on the first three word lists and the fourth and final word list for the PI condition, with Word list (1, 2, 3, 4) as the independent variable and Retrieval Score as the dependent variable. The analysis showed a significant main effect of Condition, $F(1,29) = 68.279$, $p < .001$, $\eta 2 = .702$. Post-hoc comparisons using paired t-tests and Bonferroni correction for multiple comparisons showed that the retrieval scores were significantly higher for word list 1 compared to word list 2 ($p = .001$), word list 3 ($p < .001$) and word list 4 ($p < .001$), and for word list 2 compared to word list 3 ($p < .001$) and word list 4 ($p < .001$), as well as for word list 3 compared to word list 4 ($p = .025$).

S2. *Pupil change during retrieval*

A one-way repeated-measures ANOVA was conducted on the first three word lists and the fourth and final word list for the PI condition, with Word list (1, 2, 3, 4) as the independent variable and Pupil dilation (change in diameter in relation to baseline) as the dependent variable. The analysis showed a significant main effect of Condition, $F(1,29) = 7.851$, $p < .001$, $\eta 2 = .213$. Post-hoc comparisons using paired t-tests and Bonferroni correction for multiple comparisons showed that pupil diameters were significantly larger during retrieval of word list 1 compared to word list 2 ($p = .025$), word list 3 ($p = .004$) and word list 4 ($p = .008$). Differences between any combinations of word lists 2, 3 and 4 were not significant.

S3. *Pupil change during encoding*

A one-way repeated-measures ANOVA was conducted on the first three word lists and the fourth and final word list for the PI condition, with Word list (1, 2, 3, 4) as the independent variable and Pupil dilation (change in diameter in relation to baseline) as the dependent variable. The analysis showed a significant main effect of Condition, $F(1,29) = 7.432$, $p < .001$, $\eta 2 = .204$. Post-hoc comparisons using paired t-tests and Bonferroni correction for multiple comparisons showed that pupil diameters were significantly larger during retrieval of word list 2 compared to both word list 3 ($p = .001$) and word list 4 ($p = .002$). No other differences were significant. A paired t-test between PI and RPI conditions during encoding of the fourth and final word list showed no significant difference in pupil diameter ($t(29) = 0.947$, $P = 0.35$). See Figure S1A.

To investigate the temporal dynamics during encoding of the fourth and final word list we also conducted permutation tests (20 000 permutations) on the per participant and condition (PI and RPI) average pupil signal. To control for multiple comparisons in time series data, significant differences between conditions were determined using the t_{max} method[1]. The analysis revealed that PI and RPI conditions did not differ significantly in any time point during the encoding of the three words (see Figure S1B).

Supplementary material

S4. *Semantic categories*

The following 36 semantic categories were selected from the Swedish category norms developed by Hellerstedt, Rasmussen and Johansson[2] (here translated into English):

Non-alcoholic beverages, Flowers, Musical Instruments, Metals, Dances, Swedish provinces, Dogs, Fishes, Sports, Relatives, Units of measure, Car brands, Footwear, Colors, Birds, Units of time, Cartoons, Countries, Textiles, A means of transport, American states, Furniture, Musicians, Toys, Sciences, Professions, Body parts, Something to read, Tools, Spices, Insects, Cities, Shapes of nature, Weathers, Military ranks, A type of boat.

Supplementary figures

a **b**

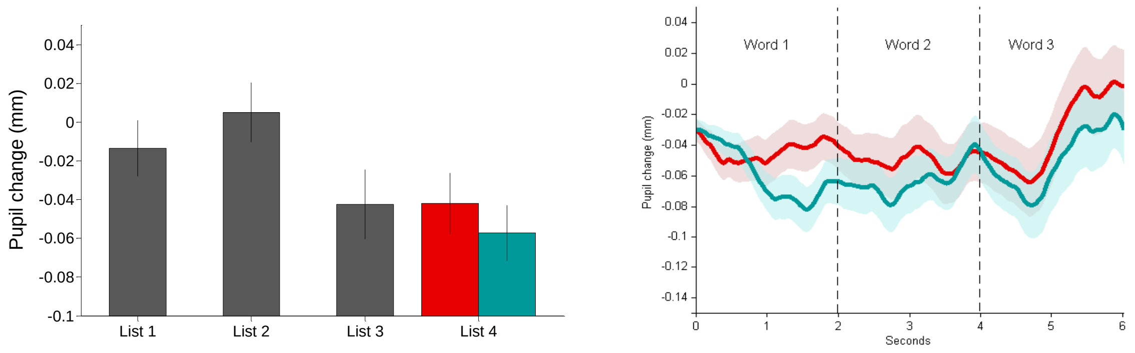

Figure S1. Pupillary changes during encoding. (**a**) Average pupil change in mm (compared to baseline) for word lists 1-3 and for the fourth and final word list when separated into PI and RPI conditions. Error bars denote SEM. (**b**) Aggregated stimulus-locked pupil change in mm during encoding of the fourth and final word list for PI and RPI conditions. The dotted lines represent onset and offset of the three words. Shaded areas for both PI and RPI conditions denote SEM.

a **b**

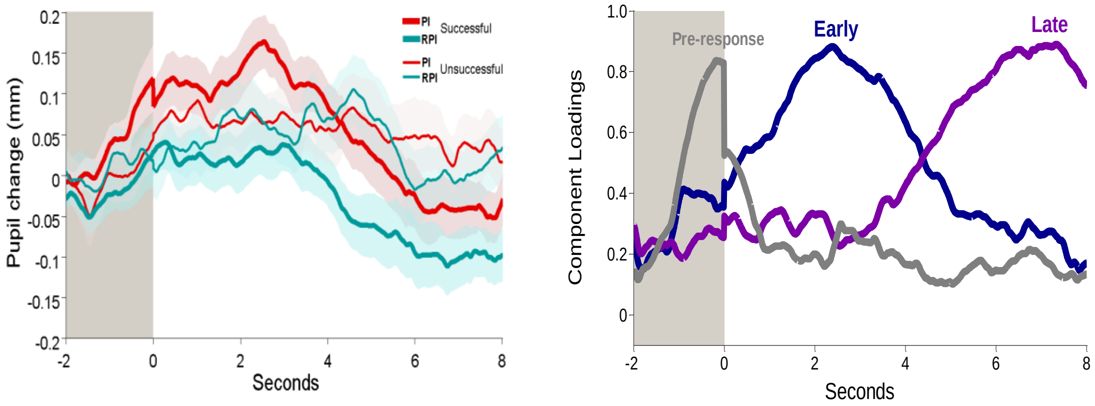

Figure S2. Pupillary changes during successful and unsuccessful retrieval attempts. (**a**) Aggregated stimulus-locked pupil change in mm during retrieval of the fourth and final word list for PI and RPI conditions over Successful and Unsuccessful retrieval. The shaded grey area to the left in the figure represents the initiation phase (2 seconds), i.e. before participants were allowed to respond. Shaded areas for both PI and RPI conditions denote SEM. (**b**) Principal component analysis (PCA) on the pupil data. Displayed are standardized component loadings for the 3 components that accounted for the majority of the variance (67.9 %): Pre-response Component (8,7 %), Early Component (28.0 %) and Late Component (31.2 %).

Supplementary references

1. Blair, R.C., and Karniski, W. (1993). An alternative method for significance testing of waveform difference potentials. *Psychophysiology* **30**, 518-524.
2. Hellerstedt, R., Rasmussen, A., & Johansson, M. Swedish category norms. *Lund Psychol. Rep.* 12, 1-96 (2012).
